# 2,5,7-Trimethyl-3-phenyl­sulfinyl-1-benzo­furan

**DOI:** 10.1107/S1600536808019302

**Published:** 2008-07-05

**Authors:** Hong Dae Choi, Pil Ja Seo, Byeng Wha Son, Uk Lee

**Affiliations:** a Department of Chemistry, Dongeui University, San 24 Kaya-dong, Busanjin-gu, Busan 614-714, Republic of Korea; b Department of Chemistry, Pukyong National University, 599-1 Daeyeon 3-dong, Nam-gu, Busan 608-737, Republic of Korea

## Abstract

The title compound, C_17_H_16_O_2_S, was prepared by the oxidation of 2,5,7-trimethyl-3-phenyl­sulfanyl-1-benzofuran with 3-chloro­peroxy­benzoic acid. The O atom and the phenyl group of the phenyl­sulfinyl substituent lie on opposite sides of the plane of the benzofuran fragment. The phenyl ring is nearly perpendicular to the plane of the benzofuran unit [88.30 (9)°] and is tilted slightly towards it. No π–π or C—H⋯π inter­actions are observed between neighbouring mol­ecules in the crystal structure because of steric hindrance induced by the three methyl groups.

## Related literature

For the crystal structures of similar 3-phenyl­sulfinyl-1-benzofuran derivatives, see: Choi *et al.* (2007 ▶, 2008 ▶).
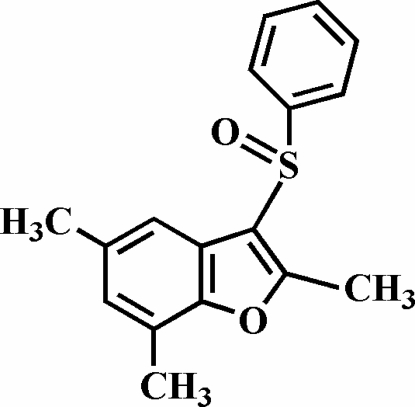


## Experimental

### 

#### Crystal data

C_17_H_16_O_2_S
*M* *_r_* = 284.36
Monoclinic, 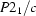

*a* = 18.393 (2) Å
*b* = 6.1515 (6) Å
*c* = 13.054 (1) Å
β = 93.024 (2)°
*V* = 1474.9 (2) Å^3^
*Z* = 4
Mo *K*α radiation
μ = 0.22 mm^−1^
*T* = 298 (2) K
0.20 × 0.10 × 0.10 mm

#### Data collection

Bruker SMART CCD diffractometer
Absorption correction: none
8615 measured reflections
3215 independent reflections
1611 reflections with *I* > 2σ(*I*)
*R*_int_ = 0.066

#### Refinement

*R*[*F* ^2^ > 2σ(*F* ^2^)] = 0.054
*wR*(*F* ^2^) = 0.163
*S* = 1.01
3215 reflections
184 parameters
H-atom parameters constrained
Δρ_max_ = 0.17 e Å^−3^
Δρ_min_ = −0.20 e Å^−3^

### 

Data collection: *SMART* (Bruker, 2001 ▶); cell refinement: *SAINT* (Bruker, 2001 ▶); data reduction: *SAINT*; program(s) used to solve structure: *SHELXS97* (Sheldrick, 2008 ▶); program(s) used to refine structure: *SHELXL97* (Sheldrick, 2008 ▶); molecular graphics: *ORTEP-3* (Farrugia, 1997 ▶) and *DIAMOND* (Brandenburg, 1998 ▶); software used to prepare material for publication: *SHELXL97*

## Supplementary Material

Crystal structure: contains datablocks global, I. DOI: 10.1107/S1600536808019302/rn2045sup1.cif

Structure factors: contains datablocks I. DOI: 10.1107/S1600536808019302/rn2045Isup2.hkl

Additional supplementary materials:  crystallographic information; 3D view; checkCIF report

## Figures and Tables

**Fig. 1.**
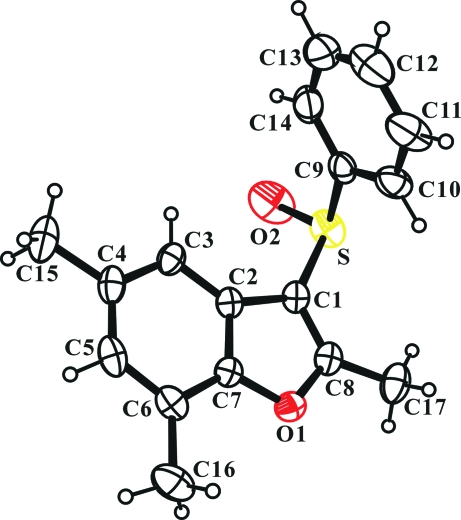
The molecular structure of the title compound, showing displacement ellipsoides drawn at the 30% probability level.

## supplementary crystallographic information

### Comment

This work is related to the our communications on the synthesis and structures of 3-phenylsulfinyl-1-benzofuran analogues, *viz*. 2,5-dimethyl-3-phenylsulfinyl-1-benzofuran (Choi *et al.*, 2007) and 2,4,6,7-tetramethyl-3-phenylsulfinyl-1-benzofuran (Choi *et al.*, 2008). Here we report the crystal structure of the title compound, 2,5,7-trimethyl-3-phenylsulfinyl-1-benzofuran (Fig. 1).

The benzofuran unit is essentially planar, with a mean deviation of 0.007 (2) Å from the least-squares plane defined by the nine constituent atoms. The phenyl ring (C9—C14) is almost perpendicular to the plane of the benzofuran ring system [88.30 (9)°] and is tilted slightly towards it. In the crystal structure, π—π or C—H···π interactions between adjacent molecules are prevented by the steric influence of the three methyl groups in the molecule.

### Experimental

77% 3-chloroperoxybenzoic acid (247 mg, 1.1 mmol) was added in small portions to a stirred solution of 2,5,7-trimethyl-3-phenylsulfanyl-1-benzofuran (268 mg, 1.0 mmol) in dichloromethane (20 ml) at 273 K. After being stirred at room temperature for 2 h, the mixture was washed with saturated sodium bicarbonate solution and the organic layer was separated, dried over magnesium sulfate, filtered and concentrated under vacuum. The residue was purified by column chromatography (hexane-ethyl acetate, 1:1 *v*/*v*) to afford the title compound as a colorless solid [yield 82%, m.p. 393–394 K; *R*_f_ = 0.65 (hexane-ethyl acetate, 1:1 *v*/*v*)]. Single crystals suitable for X-ray diffraction were prepared by evaporation of a solution of the title compound in acetone at room temperature. Spectroscopic analysis: ^1^H NMR (CDCl_3_, 400 MHz) δ 2.22 (s, 3H), 2.41 (s, 3H), 2.75 (s, 3H), 6.84 (d, J = 6.96 Hz, 2H), 7.43–7.51 (m, 3H), 7.67 (d, J = 6.60 Hz, 2H); EI—MS 284 [*M*^+^].

### Refinement

All H atoms were positioned geometrically and refined using a riding model, with C—H = 0.93 Å for aromatic H atoms and 0.96 Å for methyl H atoms, respectively, and with *U*_iso_(H) = 1.2Ueq(C) for aromatic and 1.5Ueq(C) for methyl H atoms.

### Crystal data

**Table:**

C_17_H_16_O_2_S	*F*_000_ = 600
*M**_r_* = 284.36	*D*_x_ = 1.281 Mg m^−^^3^
Monoclinic, *P*2_1_/*c*	Mo *K*α radiation λ = 0.71073 Å
Hall symbol: -P_2ybc	Cell parameters from 1802 reflections
*a* = 18.393 (2) Å	θ = 3.1–24.3º
*b* = 6.1515 (6) Å	µ = 0.22 mm^−^^1^
*c* = 13.054 (1) Å	*T* = 298 (2) K
β = 93.024 (2)º	Block, colorless
*V* = 1474.9 (2) Å^3^	0.20 × 0.10 × 0.10 mm
*Z* = 4	

### Data collection

**Table:**

Bruker SMART CCD diffractometer	3215 independent reflections
Radiation source: fine-focus sealed tube	1611 reflections with *I* > 2σ(*I*)
Monochromator: graphite	*R*_int_ = 0.066
Detector resolution: 10.0 pixels mm^-1^	θ_max_ = 27.0º
*T* = 298(2) K	θ_min_ = 1.1º
φ and ω scans	*h* = −18→23
Absorption correction: none	*k* = −6→7
8615 measured reflections	*l* = −16→13

### Refinement

**Table:**

Refinement on *F*^2^	Secondary atom site location: difference Fourier map
Least-squares matrix: full	Hydrogen site location: inferred from neighbouring sites
*R*[*F*^2^ > 2σ(*F*^2^)] = 0.054	H-atom parameters constrained
*wR*(*F*^2^) = 0.163	*w* = 1/[σ^2^(*F*_o_^2^) + (0.0668*P*)^2^ + 0.2115*P*] where *P* = (*F*_o_^2^ + 2*F*_c_^2^)/3
*S* = 1.01	(Δ/σ)_max_ < 0.001
3215 reflections	Δρ_max_ = 0.17 e Å^−^^3^
184 parameters	Δρ_min_ = −0.20 e Å^−^^3^
Primary atom site location: structure-invariant direct methods	Extinction correction: none

### Special details

**Table:**

Geometry. All e.s.d.'s (except the e.s.d. in the dihedral angle between two l.s. planes) are estimated using the full covariance matrix. The cell e.s.d.'s are taken into account individually in the estimation of e.s.d.'s in distances, angles and torsion angles; correlations between e.s.d.'s in cell parameters are only used when they are defined by crystal symmetry. An approximate (isotropic) treatment of cell e.s.d.'s is used for estimating e.s.d.'s involving l.s. planes.
Refinement. Refinement of *F*^2^ against ALL reflections. The weighted *R*-factor *wR* and goodness of fit *S* are based on *F*^2^, conventional *R*-factors *R* are based on *F*, with *F* set to zero for negative *F*^2^. The threshold expression of *F*^2^ > σ(*F*^2^) is used only for calculating *R*-factors(gt) *etc*. and is not relevant to the choice of reflections for refinement. *R*-factors based on *F*^2^ are statistically about twice as large as those based on *F*, and *R*- factors based on ALL data will be even larger.

### Fractional atomic coordinates and isotropic or equivalent isotropic displacement parameters (Å^2^)

**Table:**

	*x*	*y*	*z*	*U*_iso_*/*U*_eq_	
S	0.33491 (5)	0.75793 (15)	0.41600 (7)	0.0730 (3)	
O1	0.12972 (11)	0.7187 (3)	0.31945 (14)	0.0536 (5)	
O2	0.34960 (15)	0.7328 (5)	0.52790 (19)	0.1133 (11)	
C1	0.24341 (15)	0.6884 (5)	0.3874 (2)	0.0481 (7)	
C2	0.20160 (15)	0.5094 (4)	0.42636 (19)	0.0438 (7)	
C3	0.21511 (17)	0.3364 (5)	0.4936 (2)	0.0517 (7)	
H3	0.2611	0.3158	0.5251	0.062*	
C4	0.15868 (19)	0.1960 (5)	0.5125 (2)	0.0577 (8)	
C5	0.09051 (19)	0.2308 (5)	0.4641 (2)	0.0653 (9)	
H5	0.0534	0.1338	0.4775	0.078*	
C6	0.07447 (16)	0.4016 (5)	0.3972 (2)	0.0571 (8)	
C7	0.13246 (15)	0.5374 (4)	0.38166 (19)	0.0450 (7)	
C8	0.19788 (17)	0.8067 (5)	0.3253 (2)	0.0517 (7)	
C9	0.37498 (15)	0.5292 (5)	0.3559 (2)	0.0552 (8)	
C10	0.36512 (17)	0.5067 (6)	0.2517 (3)	0.0727 (10)	
H10	0.3375	0.6073	0.2134	0.087*	
C11	0.3964 (2)	0.3347 (9)	0.2050 (3)	0.0974 (14)	
H11	0.3891	0.3161	0.1345	0.117*	
C12	0.4385 (2)	0.1893 (8)	0.2609 (5)	0.1024 (14)	
H12	0.4588	0.0715	0.2281	0.123*	
C13	0.4510 (2)	0.2152 (7)	0.3637 (4)	0.0973 (14)	
H13	0.4807	0.1179	0.4009	0.117*	
C14	0.41888 (19)	0.3882 (7)	0.4128 (3)	0.0779 (11)	
H14	0.4270	0.4084	0.4831	0.094*	
C15	0.1696 (2)	0.0106 (6)	0.5874 (3)	0.0864 (11)	
H15A	0.2207	−0.0077	0.6043	0.130*	
H15B	0.1503	−0.1207	0.5569	0.130*	
H15C	0.1449	0.0422	0.6486	0.130*	
C16	0.00061 (18)	0.4398 (7)	0.3461 (3)	0.0873 (12)	
H16A	−0.0161	0.5825	0.3631	0.131*	
H16B	−0.0329	0.3332	0.3695	0.131*	
H16C	0.0035	0.4280	0.2731	0.131*	
C17	0.2083 (2)	1.0024 (5)	0.2620 (2)	0.0760 (10)	
H17A	0.2548	1.0662	0.2803	0.114*	
H17B	0.1704	1.1056	0.2737	0.114*	
H17C	0.2063	0.9624	0.1908	0.114*	

### Atomic displacement parameters (Å^2^)

**Table:**

	*U*^11^	*U*^22^	*U*^33^	*U*^12^	*U*^13^	*U*^23^
S	0.0663 (6)	0.0763 (7)	0.0762 (6)	−0.0265 (5)	0.0011 (4)	−0.0218 (5)
O1	0.0570 (13)	0.0537 (13)	0.0496 (12)	0.0046 (10)	−0.0005 (9)	−0.0029 (10)
O2	0.0903 (18)	0.178 (3)	0.0700 (17)	−0.0144 (18)	−0.0154 (14)	−0.0556 (17)
C1	0.0577 (18)	0.0457 (17)	0.0409 (16)	−0.0104 (14)	0.0017 (13)	−0.0058 (12)
C2	0.0541 (17)	0.0419 (16)	0.0356 (14)	−0.0058 (13)	0.0054 (12)	−0.0090 (12)
C3	0.067 (2)	0.0490 (17)	0.0387 (16)	0.0014 (15)	0.0030 (14)	−0.0008 (13)
C4	0.082 (2)	0.0468 (19)	0.0457 (18)	−0.0085 (16)	0.0177 (16)	−0.0021 (13)
C5	0.073 (2)	0.061 (2)	0.064 (2)	−0.0234 (18)	0.0229 (17)	−0.0057 (17)
C6	0.0527 (19)	0.064 (2)	0.0554 (19)	−0.0108 (16)	0.0085 (15)	−0.0140 (16)
C7	0.0528 (18)	0.0443 (17)	0.0379 (15)	−0.0011 (14)	0.0025 (13)	−0.0060 (13)
C8	0.069 (2)	0.0433 (18)	0.0434 (16)	−0.0031 (15)	0.0072 (14)	−0.0056 (13)
C9	0.0415 (17)	0.072 (2)	0.0522 (18)	−0.0146 (15)	0.0024 (14)	0.0029 (16)
C10	0.051 (2)	0.108 (3)	0.058 (2)	0.0070 (19)	−0.0011 (16)	−0.012 (2)
C11	0.061 (2)	0.144 (4)	0.088 (3)	0.006 (3)	0.006 (2)	−0.033 (3)
C12	0.082 (3)	0.096 (3)	0.132 (4)	−0.004 (3)	0.038 (3)	−0.022 (3)
C13	0.074 (3)	0.089 (3)	0.131 (4)	0.008 (2)	0.023 (3)	0.033 (3)
C14	0.067 (2)	0.100 (3)	0.067 (2)	−0.013 (2)	0.0089 (19)	0.018 (2)
C15	0.132 (3)	0.063 (2)	0.066 (2)	−0.006 (2)	0.026 (2)	0.0133 (18)
C16	0.054 (2)	0.107 (3)	0.100 (3)	−0.014 (2)	0.001 (2)	−0.017 (2)
C17	0.112 (3)	0.052 (2)	0.065 (2)	−0.0031 (19)	0.015 (2)	0.0073 (16)

### Geometric parameters (Å, °)

**Table:**

S—O2	1.480 (3)	C9—C14	1.376 (4)
S—C1	1.757 (3)	C10—C11	1.364 (5)
S—C9	1.789 (3)	C10—H10	0.9300
O1—C8	1.364 (3)	C11—C12	1.368 (6)
O1—C7	1.379 (3)	C11—H11	0.9300
C1—C8	1.347 (4)	C12—C13	1.360 (6)
C1—C2	1.450 (4)	C12—H12	0.9300
C2—C7	1.382 (4)	C13—C14	1.390 (6)
C2—C3	1.393 (4)	C13—H13	0.9300
C3—C4	1.383 (4)	C14—H14	0.9300
C3—H3	0.9300	C15—H15A	0.9600
C4—C5	1.390 (5)	C15—H15B	0.9600
C4—C15	1.509 (4)	C15—H15C	0.9600
C5—C6	1.388 (4)	C16—H16A	0.9600
C5—H5	0.9300	C16—H16B	0.9600
C6—C7	1.378 (4)	C16—H16C	0.9600
C6—C16	1.500 (4)	C17—H17A	0.9600
C8—C17	1.479 (4)	C17—H17B	0.9600
C9—C10	1.369 (4)	C17—H17C	0.9600
			
O2—S—C1	107.90 (15)	C11—C10—H10	120.5
O2—S—C9	107.03 (16)	C9—C10—H10	120.5
C1—S—C9	97.32 (13)	C10—C11—C12	120.6 (4)
C8—O1—C7	106.4 (2)	C10—C11—H11	119.7
C8—C1—C2	107.3 (2)	C12—C11—H11	119.7
C8—C1—S	123.7 (2)	C13—C12—C11	120.8 (4)
C2—C1—S	129.0 (2)	C13—C12—H12	119.6
C7—C2—C3	119.4 (3)	C11—C12—H12	119.6
C7—C2—C1	104.5 (2)	C12—C13—C14	119.4 (4)
C3—C2—C1	136.1 (3)	C12—C13—H13	120.3
C4—C3—C2	118.6 (3)	C14—C13—H13	120.3
C4—C3—H3	120.7	C9—C14—C13	119.0 (4)
C2—C3—H3	120.7	C9—C14—H14	120.5
C3—C4—C5	119.4 (3)	C13—C14—H14	120.5
C3—C4—C15	120.8 (3)	C4—C15—H15A	109.5
C5—C4—C15	119.8 (3)	C4—C15—H15B	109.5
C6—C5—C4	124.0 (3)	H15A—C15—H15B	109.5
C6—C5—H5	118.0	C4—C15—H15C	109.5
C4—C5—H5	118.0	H15A—C15—H15C	109.5
C7—C6—C5	114.3 (3)	H15B—C15—H15C	109.5
C7—C6—C16	122.0 (3)	C6—C16—H16A	109.5
C5—C6—C16	123.7 (3)	C6—C16—H16B	109.5
C6—C7—O1	124.9 (3)	H16A—C16—H16B	109.5
C6—C7—C2	124.4 (3)	C6—C16—H16C	109.5
O1—C7—C2	110.7 (2)	H16A—C16—H16C	109.5
C1—C8—O1	111.1 (2)	H16B—C16—H16C	109.5
C1—C8—C17	132.9 (3)	C8—C17—H17A	109.5
O1—C8—C17	115.9 (3)	C8—C17—H17B	109.5
C10—C9—C14	121.1 (3)	H17A—C17—H17B	109.5
C10—C9—S	118.6 (3)	C8—C17—H17C	109.5
C14—C9—S	120.2 (3)	H17A—C17—H17C	109.5
C11—C10—C9	119.1 (4)	H17B—C17—H17C	109.5
			
O2—S—C1—C8	134.5 (3)	C3—C2—C7—C6	−1.3 (4)
C9—S—C1—C8	−114.9 (3)	C1—C2—C7—C6	179.7 (3)
O2—S—C1—C2	−42.3 (3)	C3—C2—C7—O1	178.9 (2)
C9—S—C1—C2	68.3 (3)	C1—C2—C7—O1	−0.1 (3)
C8—C1—C2—C7	0.5 (3)	C2—C1—C8—O1	−0.6 (3)
S—C1—C2—C7	177.7 (2)	S—C1—C8—O1	−178.05 (18)
C8—C1—C2—C3	−178.3 (3)	C2—C1—C8—C17	−178.2 (3)
S—C1—C2—C3	−1.1 (5)	S—C1—C8—C17	4.4 (5)
C7—C2—C3—C4	0.8 (4)	C7—O1—C8—C1	0.6 (3)
C1—C2—C3—C4	179.4 (3)	C7—O1—C8—C17	178.6 (2)
C2—C3—C4—C5	0.1 (4)	O2—S—C9—C10	176.0 (2)
C2—C3—C4—C15	−177.9 (3)	C1—S—C9—C10	64.7 (3)
C3—C4—C5—C6	−0.5 (5)	O2—S—C9—C14	−8.4 (3)
C15—C4—C5—C6	177.5 (3)	C1—S—C9—C14	−119.7 (3)
C4—C5—C6—C7	0.0 (4)	C14—C9—C10—C11	3.5 (5)
C4—C5—C6—C16	−179.3 (3)	S—C9—C10—C11	179.0 (3)
C5—C6—C7—O1	−179.3 (2)	C9—C10—C11—C12	−1.5 (6)
C16—C6—C7—O1	0.0 (4)	C10—C11—C12—C13	−1.1 (7)
C5—C6—C7—C2	0.9 (4)	C11—C12—C13—C14	1.8 (6)
C16—C6—C7—C2	−179.8 (3)	C10—C9—C14—C13	−2.8 (5)
C8—O1—C7—C6	180.0 (3)	S—C9—C14—C13	−178.2 (3)
C8—O1—C7—C2	−0.2 (3)	C12—C13—C14—C9	0.2 (6)
